# Too Wet, Too Dry? Influence of Water Contamination and Overdrying on the Bond Strength of a Universal Adhesive to Dentin

**DOI:** 10.3290/j.jad.c_2521

**Published:** 2026-03-27

**Authors:** Malte Peters, Carolin Mempel, Kirstin Vach, Silke Jacker-Guhr, Nadine Schlueter, Anne-Katrin Luehrs

**Affiliations:** a Malte Peters* Doctoral Student, Department of Conservative Dentistry, Periodontology and Preventive Dentistry, Hanover Medical School, Hannover, Germany. Performed the μTBS test, wrote the manuscript, created the tables and evaluated the statistical analysis in fulfillment of the requirements for the degree “Dr. med. dent.”; b Carolin Mempel* Research Associate, Department of Conservative Dentistry, Periodontology and Preventive Dentistry, Hannover Medical School, Hannover, Germany. Performed the contact angle measurement, co-wrote and proofread the manuscript.; c Kirstin Vach Statistician and Research Associate, Department of Conservative Dentistry, Periodontology and Preventive Dentistry, Hannover Medical School, Hannover, Germany. Performed the statistical analysis, created the figures in part, co-wrote the results section and proofread the manuscript.; d Silke Jacker-Guhr Research Associate, Department of Conservative Dentistry, Periodontology and Preventive Dentistry, Hannover Medical School, Hannover, Germany. Supervised the experiments in part and proofread the manuscript.; e Nadine Schlueter Professor and Department Head, Department of Conservative Dentistry, Periodontology and Preventive Dentistry, Hannover Medical School, Hannover, Germany. Study concept and proofread the manuscript.; f Anne-Katrin Luehrs Professor, Department of Conservative Dentistry, Periodontology and Preventive Dentistry, Hannover Medical School, Hannover, Germany. Research idea and study concept, supervised the experiments in part, co-wrote paper, created the figures in part and proofread the manuscript. *Malte Peters and Carolin Mempel contributed equally to this study.

**Keywords:** contact angle, dentin surface wetness, microtensile bond strength, self-etch, etch&rinse, universal adhesive

## Abstract

**Purpose:**

The aim of this *in vitro* study was to measure the microtensile bond strength (μTBS) and the contact angle of a universal adhesive (Prime & Bond Active) to overdried and water-contaminated human dentin.

**Materials and Methods:**

After exposing flat dentin surfaces (5×5 mm^2^) of 60 caries- and restoration-free molars, test groups were either overdried or water-contaminated before adhesive application (self-etch) or after phosphoric acid etching (etch&rinse). In control groups, the adhesive was applied according to the manufacturer’s instructions. μTBS (n = 45 samples) was analyzed (24 h/thermocycling: 15,000 cycles, 5/55°C). Fracture patterns were assessed microscopically. Contact angles were measured using the sessile drop method. Statistical analysis was performed using Tobit regression and Scheffé correction for bond strength data and Kruskal–Wallis test for contact angle measurements (α = 0.05).

**Results:**

Overall, µTBS was significantly higher after etch&rinse application compared to the self-etch mode before (P < 0.001, Δ 11.04 MPa) and after aging (P < 0.001, Δ 6.73 MPa).

The highest μTBS (29.9 ± 10.4 MPa) was achieved by the etch&rinse control before aging. For etch&rinse application, water contamination and overdrying initially led to significantly lower µTBS compared to the control (P = 0.014/P = 0.007). Aging significantly decreased μTBS in both etch&rinse control and overdry groups (P < 0.001/P = 0.036). Fracture modes were predominantly adhesive (90%). Contact angle on water-contaminated dentin was significantly lower for self-etch than for etch&rinse mode (P = 0.008).

**Conclusion:**

The adhesive application mode significantly influenced bond strength. Dentin surface condition initially affected the µTBS solely in etch&rinse mode. Only on water-contaminated dentin, the contact angle was influenced by the adhesive application mode.

In modern dentistry, the market share of universal adhesives is steadily increasing; depending on the clinical indication, they can be used either in etch&rinse, selective etch, or self-etch mode.^21^ As universal adhesives achieve better bond strengths to enamel in the etch&rinse mode than in the self-etch mode, it is clinically recommended to use these systems in the selective etch mode when enamel is involved.^38^ However, the resulting rinsing and drying processes change the surface wetness of the adjacent enamel and dentin. The wetness of the dentin is decisive for establishing a sufficient adhesive layer, as a dentin surface that is too wet and one that is too dry can lead to impaired bond strength values.^55^ If dentin is conditioned and overdried, eg, by air-drying for too long after rinsing, the collagen fibrils will collapse.^35^ Depending on the adhesive system used, this can lead to a deteriorated bond strength, even if a collapsed collagen network can be partially recovered by remoistening the tooth surface and thus made accessible to adhesive penetration.^25,28^ The degree of moisture of the collagen fiber network is decisive for sufficient infiltration of the surface, depending on the adhesive system used, which still will remain technique-sensitive and therefore prone to errors.^4,20,25,49^ The interaction of universal adhesives with dentin is decisive for the long-term-stability of the bond and the avoidance of microleakage between dental hard tissues and composite. The formation of a stable hybrid layer as an interface between adhesive and dentin depends on the effective infiltration of the monomer into the demineralized collagen network and subsequent polymerization.^34^ In addition, the cavity geometries in clinical situations are overly complex, with very dry and very wet regions in a cavity at the same time, thereby making it difficult to reach perfect surface wetness.^4^ In addition to the monomers contained in the adhesive system, the solvent is also decisive for establishing dentin bonding via the formation of a hybrid layer. Universal adhesives contain a high proportion of water (up to 25%) to enable ionization of the acidic functional monomers, while at the same time they contain ethanol or acetone as solvents to accelerate water displacement.^10,44^

Although the surface wetness of dentin plays a crucial role in the establishment of the bond, only a few studies have investigated universal adhesives at diverse levels of dentin surface wetness. Therefore, the aim of this *in vitro* study was to examine the influence of dentin surface wetness on the bond strength and wetting ability of a universal adhesive. The following null hypotheses were investigated:

1. The bond strength values of the universal adhesive to dentin in self-etch and etch&rinse mode are not affected by water contamination or overdrying.
2. Aging does not affect the adhesion values.
3. The bond strength of the universal adhesive does not vary between self-etch and etch&rinse mode.
4. The contact angle does not differ between self-etch and etch&rinse mode depending on the surface wetness.

## MATERIALS AND METHODS

### Sample Size Calculation

Sample size calculation was performed with the following parameters: a group difference of 5 MPa can be demonstrated with 39 samples per sub-group, assuming that the standard deviations correspond to approximately half of the respective group mean, with a power of 90 % and an alpha of 5 % using a two-sided t-test. To compensate for possible sample exclusion due to manipulation errors, 45 samples per sub-group were used.

### Microtensile Bond Strength

In this *in vitro* study, the bond strength of a universal adhesive to human dentin was investigated after surface-contamination with water and after overdrying with an air stream. In the self-etch mode, either water contamination or overdrying took place before the adhesive system was applied, in the etch&rinse mode it was performed after phosphoric acid conditioning. All materials and their application are shown in Table 1.

**Table 1:** Materials and manufacturers, components, LOT-No. and their application

Material	Manufacturer	Components	LOT-No.	Application
**Prime & Bond Active**	Dentsply Sirona, York, USA	Bisacrylamide 1, propan-2-ol, 10-Methacryloyl-oxydecyl-dihydrogenphosphate, Bisacrylamide 2, Dipentaerythritol pentaacrylate phosphate, 4-(dimethylamino)benzonitrile	LOT-No. 1606000817 EXP: 2021-05	Application of a defined volume (15 μl) to the surface with a pipette, active application by rubbing the adhesive system onto the surface for 20 s, evaporation of the solvent with air flow for 5 s, polymerization for 10 s.
**DeTrey Conditioner 36**	Dentsply Sirona, York, USA	Phosphoric acid (36%)	LOT-No. 1909000222 EXP: 2024-08	Conditioning of the dentin for 15 s, rinsing, removal of excess water with a cloth.
**Filtek Supreme XTE A1 Body**	3M ESPE, Saint Paul, Minnesota, USA	Silane treated ceramic, 2-Propenoic acid, 2-methyl-, 3-(trimetoxysilyl) propyl ester, hydrolysis products with silica, Diurethane dimethacrylate, Bisphenol A polyethylene glycol diether dimethacrylate, (1methylethylidene) bis [4,1-phenyleneoxy(2-hydroxy-3,1-propanediyl)] bismethacrylate, polyethylene glycol dimethaceylate	LOT-No. N949706 EXP: 2021-01	Application of three individual layers of 2 mm each, separate polymerization of each layer.
**Distilled water**	MHH pharmacy			Application of 5 μl to the dentin surface.


For testing the microtensile bond strength, 60 caries-free human molars were used, which were stored in 0.5% N-chloramine T solution in a refrigerator at 4°C for no longer than 6 months until the test was performed. The use of extracted human teeth in the context of bond strength to dental hard tissues was approved by the ethics commission of the Hannover Medical School (no. 2092–2013).

**Fig 1a to g:**
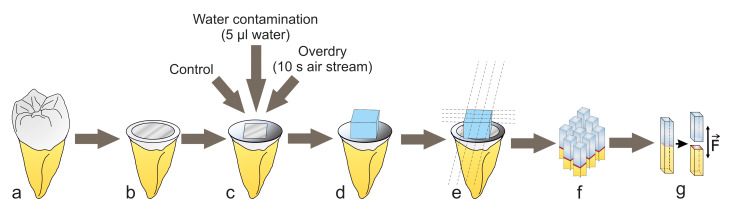
Graphical illustration of the test procedure: intact molar (a), exposed dentin surface after removal of the crown (b), isolation of a 5 × 5 mm^2^ test surface and group specific pre-treatment (c), composite build-up (three layers, each 2 mm thick, d), 4 cuts in x and y direction to obtain samples for the microtensile test (e, f), microtensile test after 24 h and after TC (15,000 cycles, 5/55°C), direction of tensile force application until fracture occurred (g).

The teeth were randomly divided into six main groups (n = 10 teeth per group) and debris was removed. All teeth were embedded in gypsum parallel to the tooth axis up to the enamel-cement interface (see Fig 1). The teeth were always kept moist during sample preparation to avoid desiccation. Using a low-speed diamond saw (Isomet Low Speed Cutter, Buehler, Esslingen, Germany), the crown was partly cut off perpendicular to the tooth axis under water cooling to expose a flat dentin surface, which was ground using abrasive sandpaper (600-grit, SiC Grinding Paper, Buehler, Esslingen, Germany) to create a clinically relevant smear layer. To achieve a controlled and standardized dentin surface for water contamination or overdrying, an area of 5 × 5 mm^2^ was demarcated using previously punched tape (Tesa insulating tape, black 10 × 15 mm^2^ 56192-10-02, Tesa SE, Norderstedt, Germany). In the etch&rinse test groups, the dentin surface was first etched with phosphoric acid for 15 s. After rinsing off the acid with water for 15 s, excess water was removed from the dentin surface with a paper towel (Tork H2, Essity Professional Hygiene Germany, Mannheim, Germany) by carefully dabbing the towel surface on the dentin for 3 s.

In the control groups, the universal adhesive was applied according to the manufacturer’s instructions (see Table 1). Per bonded area (5×5 mm^2^), 15 μl adhesive was applied using a calibrated pipette and actively spread onto the dentin surface with a microbrush. For the water contamination test groups, first 5 μl distilled water was applied, followed by the immediate application of the adhesive system (ratio of adhesive system to water: 3:1). In the overdry test groups, the dentin was overdried with a strong stream of air for 10 s before the adhesive system was applied.

After application and evaporation, the adhesive system was polymerized for 10 s with an LED polymerization lamp (Bluephase, Ivoclar Vivadent, Schaan, Liechtenstein) at a light output of >1000 mW/cm^2^. The light output of the lamp was checked and documented before each new tooth using a measuring device (Bluephase Meter, Ivoclar Vivadent). The teeth were then built up with composite (Filtek Supreme XTE A1 Body, 3M ESPE, Saint Paul, Minnesota, USA), which was applied in three layers of 2 × 5 × 5 mm^3^ (height, depth, width). Each layer was polymerized for 20 s from the occlusal side. After polymerization of the last layer, all four lateral surfaces were polymerized again for 20 s each. This resulted in a total polymerization time of 140 s.

The teeth were then cut with a low-speed diamond saw (Isomet Low Speed Cutter, Buehler, Esslingen, Germany) in x and y directions with four cuts in each direction, so that in total nine sticks (1 × 1 × 9–12 mm^3^) could be obtained (for each main group with 10 teeth: 90 sticks). Half of the sticks were stored for 24 h in distilled water at 37°C before testing, the other half of the sticks were aged for 15,000 cycles by thermocycling (5°C/55°C, 30 s dwell time, 5 s transition time each, total cycle time: 70 s, thermocycler: SD Mechatronik GmbH, Feldkirchen-Westerham, Germany) resulting in 12 sub-groups with each n = 45 sticks. Sticks that fractured during sectioning (“zero bonds”), during 24 h storage, or during TC were included in the statistics. In accordance with Armstrong et al., the values of the affected samples were set to half of the lowest value measured in the respective group.^5^ Sticks that fractured due to manipulation errors were excluded from the statistics. All sticks were measured with an electronic caliper before the experiment to determine the size of the interfacial area. To perform the μTBS test, the sticks were subjected to tensile loading (feed 0.5 mm/min) in a universal testing machine (MTD-500 plus, SD Mechatronik, Feldkirchen-Wersterham, Germany) using cyanoacrylate glue (Roxolid Aktiv-X, Hornbach, Bornheim, Germany) until fracture occurred. The maximum force (N) measured at the time of fracture was documented for each stick, then divided by the bond area (mm^2^) to calculate the bond strength in MPa.

### Fracture Analysis

Following the µTBS testing, fracture analysis was conducted using a light microscope (Stemi SV 6, Carl Zeiss Jena, Jena, Germany) at 50× magnification. Fracture types were classified as adhesive, cohesive or mixed failures.

### Contact Angle Measurements

For the contact angle measurements, 30 dentin discs (n = 30 teeth, 1 disc per tooth) were prepared using a low-speed diamond saw (Isomet Low Speed Cutter). First, the coronal part of each crown was cut as described above to expose a flat dentin surface. With a second cut parallel to the first cut, a disc with a thickness of 1–2 mm per tooth was created. All discs were then randomly divided into six groups (five discs per group) and kept moist to prevent desiccation. A standardized smear layer was created with an abrasive sandpaper (600 grit). The pretreatment of the discs for each group corresponded to the sample preparation for the μTBS test as mentioned above. In the self-etch groups, the surface was immediately rinsed with water, whereas in the etch&rinse groups, the surface was first etched with phosphoric acid. Excess water was removed from the dentin surface with a paper towel (Tork H2, Essity Professional Hygiene Germany GmbH, Mannheim, Germany) for 3 s.

In the control groups, a droplet of 6 µl of the adhesive system was dripped immediately onto the dentin surface using a calibrated pipette.

In the water contamination test groups, 2 µl water was dripped simultaneously with the adhesive system with a second calibrated pipette (this corresponds to the abovementioned ratio of adhesive system to water of 3:1 as in the μTBS testing). In the overdry test groups, the dentin was overdried with a stream of air for 10 s before the adhesive system was dropped to the surface.

For standardization, the contact angle measurements were made immediately after a 2 s waiting period with the “sessile drop method” using a Surftens universal contact angle goniometer and the respective software (Surftens 4.2, OEG, Frankfurt/Oder, Germany).

### Statistical Analyses

The data were statistically analyzed using STATA (version 17.0; College Station, TX, USA, significance level α = 0.05). Regarding microtensile bond strength data, differences in bond strength between the groups were analyzed using Tobit regression models with the group minimum values as the lower limit. Scheffé correction was used for multiple testing. For contact angle measurements, overall analysis was performed with Kruskal–Wallis test due to strong deviation from the normal distribution. Afterwards, Dunn’s test with Holm correction and Mann–Whitney U-test were used for pairwise comparisons.

## RESULTS

All results regarding microtensile bond strength (μTBS) are presented in Table 2. The Tobit regression showed significant group differences both before and after aging (P < 0.001/P < 0.001). Overall, the etch&rinse control group showed the highest μTBS (29.86 ± 10.37 MPa, Table 2).

**Table 2:** Mean values and standard deviations of microtensile bond strength (MPa, mean ± SD) of the control and test groups after 24 h of water storage and aging by thermocycling (TC)

Group	n/zero bonds/specimen excluded from statistics	Initial (24 h)	n/zero bonds/specimen excluded from statistics	After TC
Self-etch control	45/6/0	13.46 (± 8.09)	45/3/0	15.06 (± 6.99)
Self-etch water contamination	45/4/0	13.70 (± 7.88)	45/6/0	14.62 (± 8.87)
Self-etch overdry	45/4/0	16.70 (± 8.20)	45/1/0	16.25 (± 8.81)
Etch&rinse control	45/1/0	29.86 (± 10.37)	45/6/0	21.22 (± 10.77)
Etch&rinse Water contamination	45/3/0	23.13 (± 11.06)	45/6/0	24.68 (± 9.63)
Etch&rinse overdry	45/0/0	23.28 (± 8.80)	45/1/1	19.44 (± 7.97)


### Influence of the Application Mode (Self-Etch vs. Etch&Rinse)

**Fig 3:**
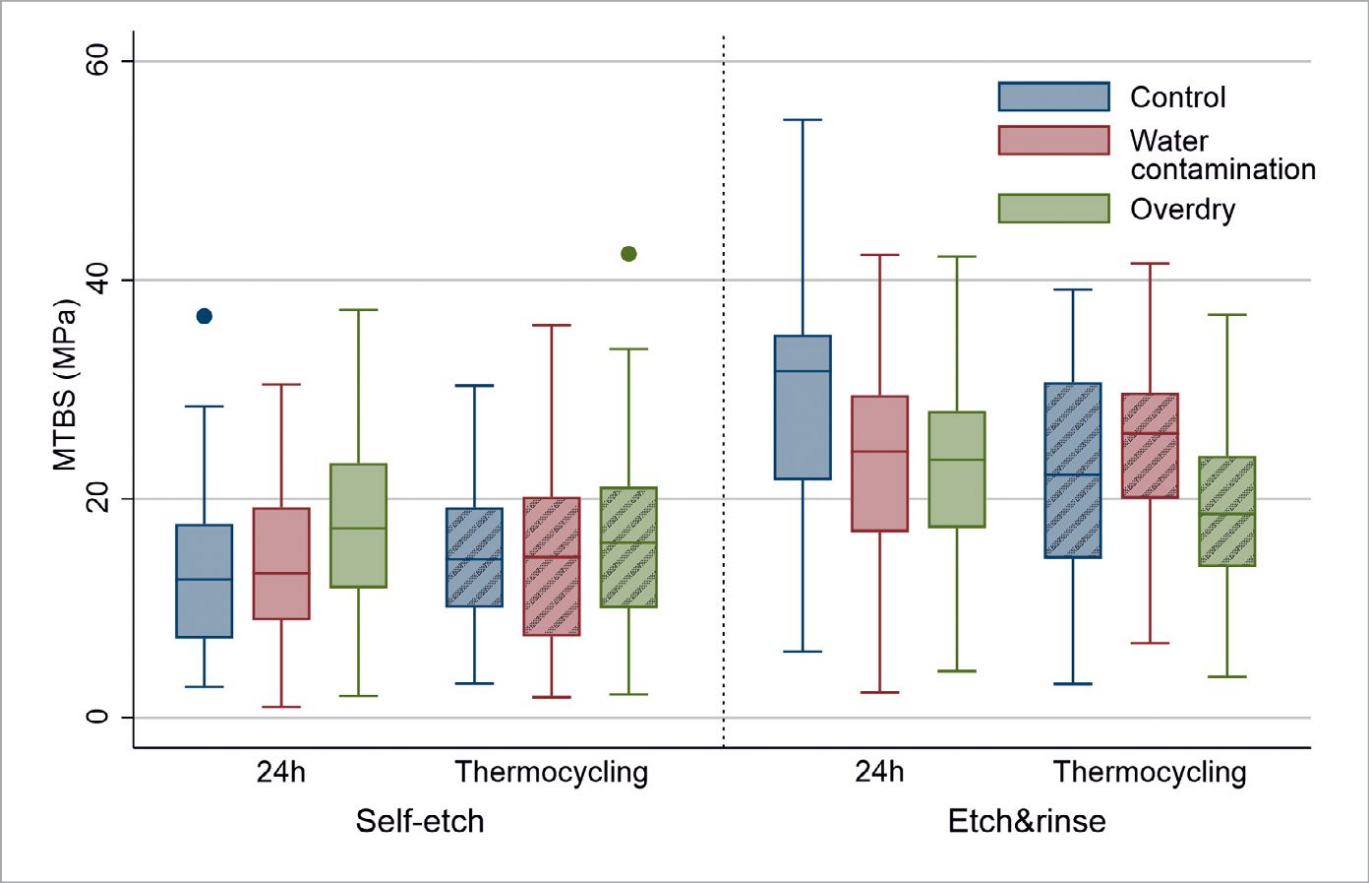
Boxplot diagram of the control and experimental groups in self-etch and etch&rinse mode before and after aging by thermocycling (crosshatched boxes) with median values of the μTBS, second and third quartiles as well as outliers (small dots).

The initial μTBS was significantly higher for the etch&rinse mode compared to the self-etch application in all groups (control, water contamination, overdry: P < 0.001/P < 0.001/P = 0.001). After TC, these differences were still present for both control and water contamination groups (P = 0.003/P < 0.001) but not for overdried dentin (P = 0.08, Table 2 and Fig 3).

When control and test groups were pooled, µTBS was significantly higher after etch&rinse application compared to the self-etch mode (P < 0.001, Δ (difference) 11.04 MPa). This difference for the pooled groups was also significant after aging (P < 0.001, Δ 6.73 MPa).

### Influence of the Dentin Surface Condition (Water Contamination vs. Overdry)

**Fig 2:**
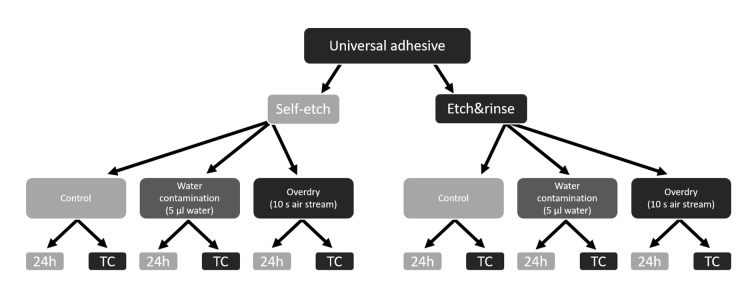
Overview of the main control and test groups, each sub-group was then tested after 24 h (water storage for 24 h at 37°C) and TC (Thermocycling (15,000 cycles, 5/55°C)).

Regarding self-etch application, Tobit regression showed no differences between control, water-contaminated or overdried dentin, both initial (P = 0.143) or after aging (P = 0.643). For etch&rinse application, μTBS was significantly influenced by the dentin surface condition before (P = 0.002) and after aging (P < 0.011). Initially, both water-contaminated and overdried dentin showed significantly lower bond strength values when compared to the control (P = 0.014/P =0.007, Table 2 and Fig 2). After aging, significant differences were only present between water-contaminated and overdried dentin (P = 0.012), but not in the control.

### Influence of Aging (24 h vs. Thermocycling)

In self-etch mode, there was no influence of artificial aging on the bond strength of the groups. In the etch&rinse mode, thermocycling led to a significant decrease in bond strength in both the control and the overdry test groups (P < 0.001/P = 0.036, Fig 3).

### Fracture Analysis

**Fig 4:**
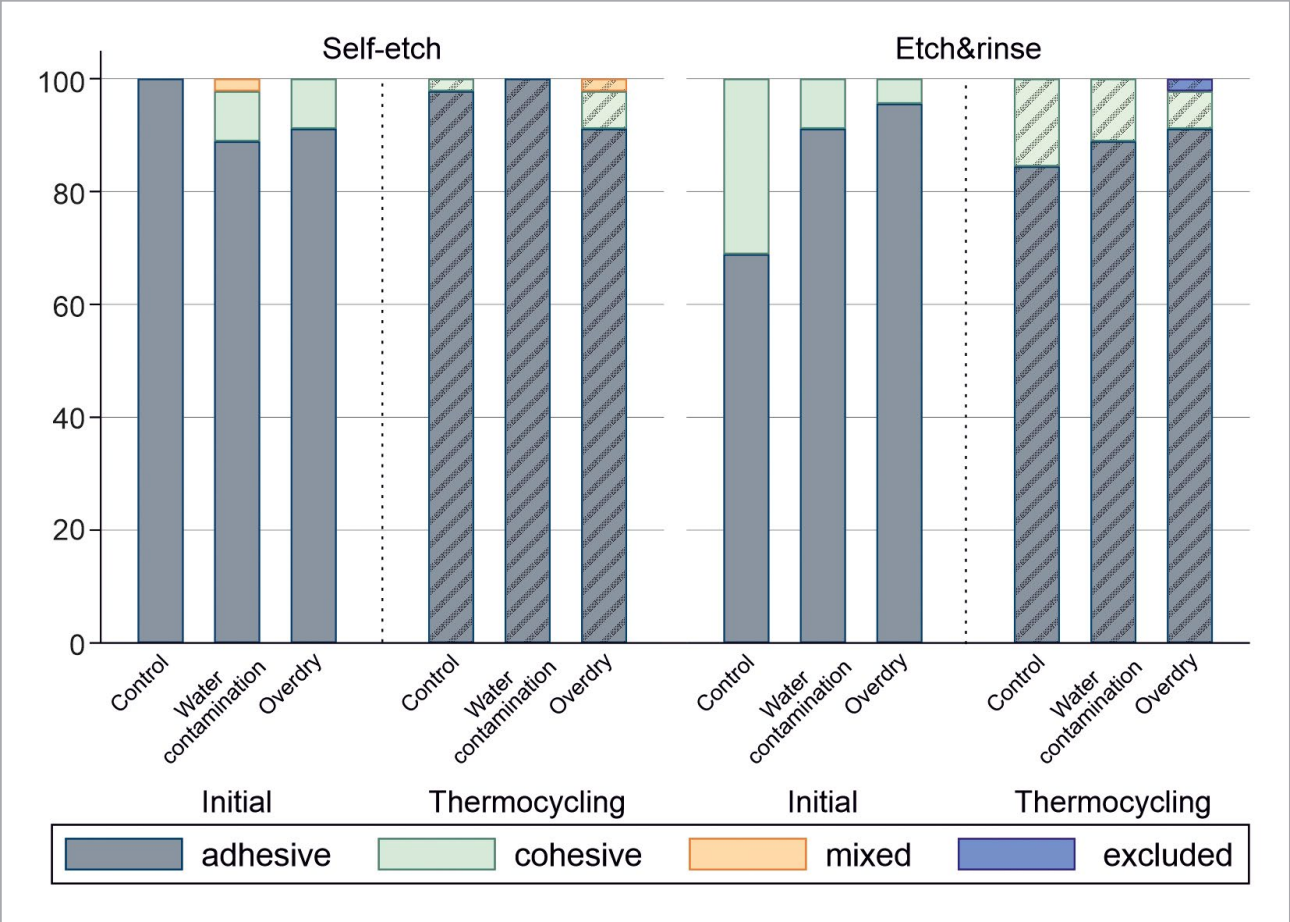
Graphical representation of the fracture modes (in %) for the control and test groups in self-etch and etch&rinse mode before and after aging by thermocycling.

The most frequently observed fracture patterns were adhesive fractures (90.91%, Fig 4). Overall, cohesive fractures occurred in 8.27% and mixed fractures in 0.37% of cases. In the etch&rinse control group before aging, which also had the highest microtensile bond strength, 31% of the fractures were cohesive. The proportion of mixed fractures was low and only present in two groups (self-etch water contamination initial and self-etch overdry thermocycling, 2% each).

### Contact Angle Measurement

**Fig 5:**
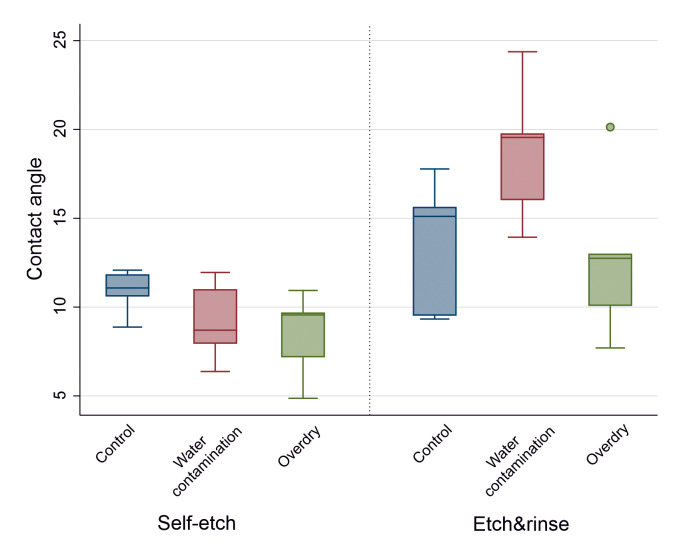
Boxplot diagram of the control and test groups in self-etch and etch&rinse mode with median values of the contact angle, second and third quartile as well as outliers (small dot).

**Fig 6:**
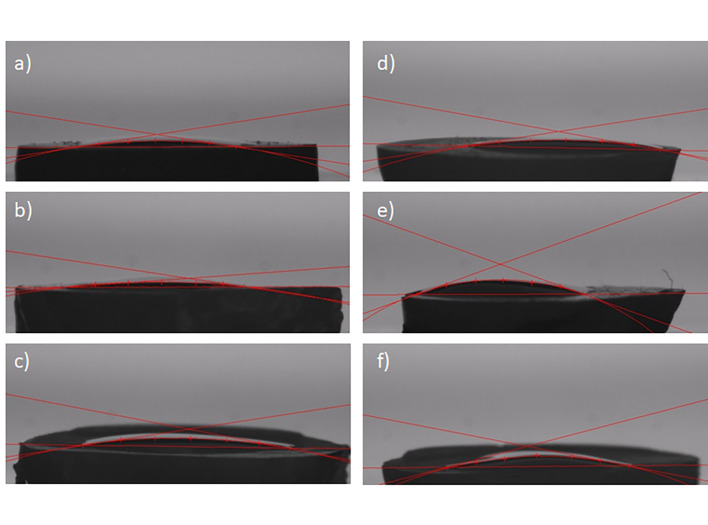
Contact angle measurements of the universal adhesive on human dentin surfaces using the “sessile drop method”: (a) self-etch control; (b) self-etch water contamination; (c) self-etch overdry;(d) etch&rinse control; (e) etch&rinse water contamination; (f) etch&rinse overdry.

The Kruskal–Wallis test showed significant differences between all groups (P = 0.017). Regarding the control groups and the overdry groups, no significant differences could be detected between the self-etch and the etch&rinse mode (P = 0.420/ P = 0.095). In contrast to that, the contact angle on water-contaminated dentin was significantly lower for self-etch than for the etch&rinse mode (P = 0.008). Within the groups where the same etch mode was applied (either self-etch or etch&rinse), no significant differences regarding the contact angle depending on surface wetness were present (P = 0.164/P = 0.112; Figs 5 and 6).

## DISCUSSION

The manufacturers’ instructions regarding moisture of the dentin surface prior to adhesive application are usually imprecise, and the amount of water residues can vary considerably depending on the cavity geometry, especially after application of the etch&rinse procedure. The results of the present study show that the bond strength of the universal adhesive investigated depends significantly on the application mode (etch&rinse > self-etch). For the etch&rinse application, water contamination and overdrying initially lowered the bond strength compared to the control, but this effect was not detectable after aging. Aging-related effects were detectable in the etch&rinse mode for the control group and overdried dentin. Regarding the contact angle, significant differences in were found in the water-contaminated and previously etched test group, which showed higher contact angles compared to the self-etch group (P = 0.008). Therefore, all null hypotheses have to be rejected.

In this study, the surface on which the water contamination or overdrying occurred was defined by a punched opening (5 × 5 mm^2^) in an adhesive tape. For water contamination, a standardized amount of water (5 μl) was used to simulate water contamination. The water contamination selected in this study corresponds to a contamination of the adhesive with 25 vol% water. This value was selected because it has been proven that phase separation occurs by a water concentration of more than 25 vol% in the adhesive mixture of BisGMA/HEMA-based adhesive systems.^45^ Although the universal adhesive investigated has a different mixture, it seems reasonable that the chemical properties with regard to phase separation are comparable. Overdry the dentin, a strong stream of air was directed onto the respective surface for 10 s. This period was chosen due to data found in the literature and is more reproducible compared to setting the endpoint for the drying time by appearance of, for example, a frosty white surface.^10,16,52^

To simulate aging, the samples in our study were subjected to a thermocycling process for 15,000 cycles, which corresponds to an intraoral retention time of 1.5 years.^17^ In this way, hydrolytic degradation and contraction or expansion stresses are induced due to the different thermal expansion behavior between the natural tooth structure and the restoration.^17,33^ In this study, thermocycling did not influence bond strength values in self-etch mode, which is consistent with results for one-step adhesive systems and universal adhesives, and partly shows a resistance against aging within the conditions applied in our study.^18,24,31,32^

The wetness of the dentin surface directly influences the interaction of adhesive systems and their individual components. In overdried dentin after previous demineralization, collapsed collagen fibrils inhibit the infiltration of low-viscosity monomers compared to a moist dentin surface.^56^ In case of water contamination, the diameter of the collagen fibrils increases due to swelling, which reduces the space for infiltration between the individual fibrils, which also leads to poorer monomer infiltration.^30^ This “water contamination” can originate from the dentinal tubules themselves, especially in dentin areas close to the pulp, or because of rinsing processes as part of the adhesive protocol.^53^ In our study, in etch&rinse application, both water-contaminated and overdried groups showed bond strength values which were significantly different from the control initially, but not after aging. It seems that on dentin pre-etched with phosphoric acid, a slight decrease in bond strength occurs in the experimental groups, but aging seems to be the main factor influencing the long-term bond strength. In the self-etch application, these effects were not present, which might prove a relative resistance of the examined universal adhesive in regard to the dentin condition. Depending on the hydrophilicity of the adhesive system, water contamination also leads to a phase separation of the hydrophobic and hydrophilic monomer components, which can result in bubble-like spaces and micelles within the hybrid layer.^48^

The consequences of such phase separation include a deterioration in polymerization and consequently a reduced bond strength.^15,50^ The increased susceptibility of one-bottle self-etch adhesives and universal adhesives to water absorption due to the hydrophilic monomers compared to multi-step adhesive systems also leads to an increased occurrence of nano-leakage after artificial aging tests.^16,22^

The extent to which the surface wetness of the dentin influences the bond strength also depends on the individual chemical composition of the adhesive system.^10^ Most universal adhesives available contain relatively viscous monomers, such as BisGMA, and low-viscosity hydrophilic co-monomers, eg, HEMA, to reduce the overall viscosity and allow diffusion into the collagen fiber network of the dentin.^9^ However, the hydrophilicity of this monomer can cause HEMA-rich universal adhesives to exhibit significantly poorer μTBS values than comparable HEMA-free universal adhesives on dentin.^42^

The universal adhesive investigated in this study neither contains molecules such as BisGMA or UDMA nor HEMA as a diluent, but instead the functional monomers 10-MDP (10-Methacryloyloxydecyl Dihydrogen Phosphate) and PENTA (Dipentaerythritol Penta-Acrylate Phosphate).^11^ Both 10-MDP and PENTA are functional monomers. 10-MDP is widely used in adhesive dentistry for bonding restorative materials like composites, ceramics, and metal/alloys to tooth structures. While 10-MDP-based adhesives generally exhibit higher immediate bonding performance, PENTA offers advantages in terms of hydrolytic stability and wetting properties.^8^ Furthermore, the adhesive system contains a newly developed molecule, the (E)-N,N’-(but-2-ene-1,4-diyl)bis(N-allylacrylamide).^11^ The molecule overcomes the surface tension of water, which means that tiny amounts of water in contact with the adhesive system do not lead to phase separation, but instead a homogeneous “adhesive-water phase” is initially formed. The adhesive system investigated in this study appears to achieve a bond comparable to that of the control group, even with increased dentin surface moisture.^41,42^ This is also confirmed by the post-aging data, as there was no significant difference between the water contamination and overdrying groups and the control in both the self-etch and etch&rinse modes.

If an adhesive bond to moist dentin has to be established, a certain amount of water is absorbed. This water can partially evaporate with the volatile solvents contained in the adhesive system, such as ethanol or, in the case of the universal adhesive investigated in our study, isopropanol, as these have a higher vapor pressure than water.^12^ However, complete evaporation of the water never occurs clinically. One way to improve water evaporation is to actively apply the adhesive system, which was carried out in our study for 20 s with a microbrush according to the manufacturer’s instructions.^43^ Another approach to accelerate the evaporation of the water is the application of warm air during the air-blowing process, but so far, this method is not investigated frequently and is rarely used in practice.^46^

Overdrying of the dentin can occur in everyday clinical practice, for example, after visualization of the enamel etching pattern when using the etch&rinse or selective etch technique. It is known from previous studies that the solvent contained in the adhesive has an influence on the bond strength to overdried dentin.^36,37^ The examined universal adhesive uses isopropanol and water as solvents instead of ethanol, allowing the potentially collapsed collagen network to re-expand after conditioning and thus enable infiltration with the formation of a hybrid layer. In earlier studies, it was already demonstrated that the bond strength to overdried dentin is not reduced, and the electron microscope revealed a homogeneous hybrid layer.^27^ These results are confirmed by our study for the self-etch application initially and after thermocycling, as well as for the aged etch&rinse groups, as the bond strength to overdried dentin did not differ significantly from the control.

In contrast to the surface wetness of the dentin, the application mode (self-etch vs etch&rinse) had a considerable influence on the bond strength values.^19,54^ Regarding the comparison of self-etch and etch&rinse procedures, previous meta-analyses on mild or ultra-mild universal adhesives either did not show any significant difference in dentin bond strength, or showed a bond strength elevation for ultra-mild adhesives.^13,38^ In our study, a universal adhesive with a pH-value of > 2.5 was used, which can be classified as “ultra mild” and might benefit from the etch&rinse approach in dentin.

Our study showed that the examined universal adhesive containing the above mentioned (E)-N,N’-(but-2-ene-1,4-diyl)bis(N-allylacrylamide) is not dependent on the moisture of the dentin surface in regard to microtensile bond strength in self-etch application. For universal adhesives, the use of the “selective enamel etching,” which limits the application of phosphoric acid to enamel margins of the cavity, is recommended in the literature, as it might improve retention rates and prevent marginal discoloration.^3,26^ Therefore, when using the selective enamel etching technique, the practitioner can also focus on drying the remaining enamel in order to visualize the respective etching pattern with air-blowing without harming the dentin bond strength of the examined adhesive. Nonetheless, the general applicability of the results should be interpreted with caution, given that only one adhesive system was evaluated.

In our study, each stick was used as a statistical unit. To avoid misleading influences on the statistical analysis, individual variability among teeth should be taken into account. Conversely, specimens derived from the same tooth may exhibit similar characteristics.^57^ Therefore, to accurately assess the relationship between microtensile bond strength and influencing factors such as tooth age, it is essential to consider clustering effects in the statistical analysis.^29^ In our study, half of the sticks were used for initial microtensile bond strength testing, while the other half were tested after aging. These sticks were consistently obtained from a defined central bonding area and were separated by tooth after each sectioning cycle, rather than being pooled and split after sectioning all teeth. Nevertheless, this procedure may be considered a limitation of the study.

The etch&rinse procedure affects the contact angle of the universal adhesive to dentin. Contact angle measurements on dentin surfaces provide information about the interaction between the adhesive and the respective surface, and it also shows the wetting ability of the adhesive depending on the dentin condition (eg, wet, dry, control).^40^

To investigate the contact angle in our study, we used the sessile drop method with direct measurement of the tangent angle at the three-phase equilibrium interfacial point.^23^ Generally, the contact angle for a good wetting ability should be smaller than 90 degrees, as it reflects the wettability and infiltration of the adhesive into the dentin surface, which applies to all examined groups.^7,23^ It is important to mention that contact angles decrease on deep dentin compared to superficial dentin.^39,51^ In our study, the interface was located mid-coronal. An etched dentin surface increases the contact angle, which was confirmed in our study for wet dentin surfaces and could have been caused by a higher dentin surface roughness after phosphoric acid application.^1,6,14,40^ The universal system analyzed is, like most universal adhesives, an unfilled adhesive.^47^ However, a comparison of six different filled and unfilled universal adhesives did not show significant differences in parameters representing the wettability, such as the contact angle.^7^ It is important to note that while contact angle provides insight into wettability, other factors such as adhesive composition, application technique, and the presence of a smear layer also play crucial roles in the overall bonding effectiveness of dental adhesives.^2^

## CONCLUSION

The etch&rinse application of the investigated universal adhesive showed significantly higher bond strength values compared to the self-etch application. Water contamination or overdrying only affects the dentin bond strength in etch&rinse application initially, but not in the long term. Artificial aging influenced the bond strength in two out of three etch&rinse groups. Contact angle measurements showed significantly higher values on wet dentin for the etch&rinse approach. In this *in vitro* setup, the application mode of the adhesive rather than the surface wetness seems to affect the dentin bond strength. Nevertheless, the results of this study have to be assessed in the context of the adhesive’s chemical composition.

### Acknowledgments

The research was funded by the financial resources of the Department of Conservative Dentistry, Periodontology and Preventive Dentistry, Hannover Medical School, Germany.

### Clinical Relevance

Independent of the dentin surface condition, etch&rinse application leads to higher dentin bond strength. In the long term, both water contamination and overdrying do not affect dentin bond strength independent of the etch mode applied.
